# HBV Reactivation in Patients with Past Infection Affected by Non-Hodgkin Lymphoma and Treated with Anti-CD20 Antibody Based Immuno-Chemotherapy: A Multicenter Experience

**DOI:** 10.3390/jpm12020285

**Published:** 2022-02-15

**Authors:** Michele Clerico, Irene Dogliotti, Paola Ghione, Vittorio Ruggero Zilioli, Francesco Merli, Barbara Botto, Wael Al Essa, Marcella Battaglini, Daniele Grimaldi, Loretta Cervi, Simone Ragaini, Simone Ferrero, Veronica Peri, Gabriele De Luca, Alfredo Marzano, Federica Cavallo

**Affiliations:** 1 Division of Hematology, Department of Molecular Biotechnology and Health Sciences, University of Torino, A.O.U. Città della Salute e della Scienza di Torino, 10126 Turin, Italy; michele.clerico@unito.it (M.C.); simone.ragaini@unito.it (S.R.); simone.ferrero@unito.it (S.F.); veronica.peri@unito.it (V.P.); gabriele.deluca@unito.it (G.D.L.); 2 Stem Cell Transplant Unit, A.O.U. Città della Salute e della Scienza di Torino, 10126 Turin, Italy; irenedogl@hotmail.com; 3 Lymphoma Program, Department of Medicine, Roswell Park Comprehensive Cancer Center, Buffalo, NY 14263, USA; paolaghi@buffalo.edu; 4 Division of Hematology, ASST Grande Ospedale Metropolitano Niguarda, 20162 Milan, Italy; vittorioruggero.zilioli@ospedaleniguarda.it; 5 Division of Hematology, Azienda Unità Sanitaria Locale—IRCCS, 42123 Reggio Emilia, Italy; francesco.merli@ausl.re.it; 6 Division of Hematology, A.O.U. Città della Salute e della Scienza di Torino, 10126 Turin, Italy; bbotto@cittadellasalute.to.it; 7 Division of Hematology, Department of Translational Medicine, University of Eastern Piedmont, 28100 Novara, Italy; wael.alessa@uniupo.it; 8 DDINOGMI, Department University of Genoa, IRCCS Istituto Giannina Gaslini, 16147 Genoa, Italy; marcellabattaglini@yahoo.it; 9 Division of Hematology, A.O.S. Croce e Carle, 12100 Cuneo, Italy; grimaldi.d@ospedale.cuneo.it; 10 Division of Pharmacy, ASST Grande Ospedale Metropolitano Niguarda, 20162 Milan, Italy; loretta.cervi@ospedaleniguarda.it; 11 Division of Gastroenterology and Hepatology, San Giovanni Battista Hospital, 10126 Turin, Italy; alfredomarzano@yahoo.it

**Keywords:** hepatitis B virus (HBV), non-Hodgkin lymphoma (NHL), HBV reactivation (HBVr), rituximab, lamivudine (LAM)

## Abstract

Hepatitis B virus reactivation (HBVr) can develop in HBV surface antigen (HBsAg) positive or HBsAg-negative and anti-hepatitis B core antigen antibodies (anti-HBc) positive (past HBV infection) patients receiving immuno-chemotherapy for hematological malignancies. A higher rate of HBVr is associated with the use of rituximab (R) in patients with past HBV infection, thus justifying an antiviral prophylaxis. In this study we evaluated the incidence of HBVr in a real-life cohort of 362 anti-HBc-positive subjects affected by non-Hodgkin lymphoma (NHL), mainly receiving lamivudine (LAM) prophylaxis (93%) and all undergoing a R-containing regimen. A retrospective, multicenter, observational study was conducted in 4 Italian Hematology Departments. The primary endpoint was the incidence of virologic (HBV DNA-positive), serologic (HBsAg-positive) and clinical (ALT increase > 3 × upper limit of normal) HBVr, which occurred in five, four and one patients, respectively, with a total HBVr rate of 1.4%. None of them had to discontinue the chemotherapy program, while two patients required a delay. Treatment-related adverse events (AEs) were reported during LAM prophylaxis in three patients (0.9%). In conclusion, this study confirms the efficacy and safety of LAM prophylaxis in anti-HBc-positive patients undergoing R-containing regimens.

## 1. Introduction

Despite that the prevalence of hepatitis B virus (HBV) infection has been globally decreasing during the last decades due to vaccination programs and effective treatments, it remains a major public health issue worldwide [1,2,3]: it is estimated that more than 240 millions people are chronic HBV surface antigen (HBsAg) carriers and at least two billions show hepatitis B core antigen antibodies (anti-HBc-positive—past HBV infection), with large regional variations [4].

In particular, in the past infection group, patients who require deep immunosuppressive therapy are at risk of virus reactivation, with different possible scenarios, ranging from serum HBV-DNA detection in asymptomatic patients to potentially fatal hepatitis and liver failure [5]. This event was reported in patients receiving immuno-chemotherapy protocols for hematological malignancies or undergoing hematopoietic stem cells transplantation (HSCT) [6,7,8,9,10,11]. In this context, the highest rates of reactivation are associated with the use of rituximab (R), an anti-CD20 monoclonal antibody widely used against lymphoproliferative disorders [8,10,12,13].

HBV reactivation can occur in both patients who have overt chronic infection (HBsAg-positive) and patients who have markers of past HBV infection, as evidenced by clearance of circulating HBsAg and presence of antibodies to hepatitis B core antigen (anti-HBc) with or without antibodies to hepatitis B surface antigen (anti-HBs) [8]. Among patients with past HBV, resolved HBV infection is considered if the anti-HBs is positive; if the anti-HBs is negative, then this is considered isolated anti-HBc-positive [14]. While there is wide consensus on the imperative need for screening HBV serology in all patients affected by lymphoma undergoing R-based regimens and on the importance of using prophylactic nucleos(t)ide analog treatment (NAT) in HBsAg-positive patients, there is still a lack of consensus on managing past HBV [15]. The European Association for the Study of Liver (EASL) Clinical Practice Guidelines (2017) and the recent American Society of Clinical Oncology (ASCO) statements [14] suggest the use of an anti-HBV prophylaxis in this subgroup of patients; for this purpose, lamivudine (LAM) may be used safely, but also entecavir (ETV), tenofovir disoproxil fumarate (TDF) and tenofovir alafenamide (TAF) can be considered [3]. Nevertheless, some authors still prefer to start pre-emptive NAT only if HBV reactivation is identified at an early stage by HBV DNA or HBsAg frequent monitoring [14,16,17]. In spite of this, randomized studies demonstrated that the risk of HBV reactivation under chemotherapy (CT) plus R ranges from 10.7% to 18% adopting the “pre-emptive strategy”, compared to 0% and 2.4% using prophylaxis with LAM, TDF or ETV, respectively [9,18,19]. However, the use of LAM vs TDF/ETV in this setting remains debated.

A meta-analysis including one randomized trial and several observational studies showed a pooled risk estimation of 16.9% reactivation rate (with transaminase elevation and/or detectable HBV DNA) in patients treated with R undergoing the pre-emptive strategy, thus not receiving upfront NAT [13]. In another recent meta-analysis of three prospective studies of patients who received anti-CD20 monoclonal antibodies, the pooled estimated relative risk was 0.17, indicating a significantly lower risk of HBV reactivation in the antiviral prophylaxis group [20]. Finally, in a previous Italian study, only 1 of 85 patients with past HBV infection treated with R-CT and receiving prophylactic LAM showed a serum conversion of HBsAg [19].

As a prophylaxis, LAM seems to be more widely used, thanks to its availability, low cost and safety profile, especially in Western Countries [21,22]. Moreover, nowadays, only the use of prophylactic LAM is reimbursed in hemato-oncological patients with past HBV in Italy [15,21].

The aim of this study was to evaluate the incidence of HBV reactivation in a large real-life cohort of patients with past HBV infection, mainly treated with LAM because affected by non-Hodgkin lymphoma (NHL) and undergoing a R-containing regimen. Therefore, we collected data obtained from the participating centers in order to evaluate the safety and efficacy of LAM whenever used.

## 2. Materials and Methods

### 2.1. Population

A retrospective, multicenter, observational study was conducted in four Italian Hematology Departments. The study protocol was approved by the Ethics Committees of participating centers and by the institutional review board of each hematology unit. Written informed consent was collected from all patients except for those patients who were unable to give it (according to Italian law 9/2016 Autorizzazione Generale Garante della Privacy) [23].

Adult patients (>18 years) with a diagnosis of NHL, treated at one of the participating centers with a R-based immunochemotherapy and with documented pre-treatment positivity for anti-HBc were included in the study. Chronic HBV infection (defined by the presence of HBsAg in serum) and/or detection of HBV DNA were considered exclusion criteria, as well as human immunodeficiency virus (HIV), hepatitis D virus (HDV) coinfection and a diagnosis of chronic lymphocytic leukemia (CLL).

In patients who received prophylaxis, LAM was administered at 100 mg qd from the beginning of chemoimmunotherapy and continued after the end of the regimen, as per local standard of practice. Similarly, HBV serology monitoring was variable in the respective Centers.

Data on patient characteristics and outcomes were extracted by study investigators from medical records or clinical charts, including demographic data, NHL subtype, planned NHL treatment scheme and duration, as well as HBV and hepatitis C virus (HCV) serological profile, type and duration of prophylaxis, anti-HBV treatment in case of reactivation.

HBV reactivation (HBVr) was defined as virological (HBV DNA positive in absence of HBsAg seroreversion and transaminases increase > 3 × upper limit of normal [ULN]), serological (HBsAg seroreversion) and clinical (HBV DNA positive in presence of HBsAg seroreversion and transaminases increase > 3 × ULN).

### 2.2. Endpoints

The primary endpoint of the study was the incidence of virological (HBV DNA-positivity), serological (HBsAg seroconversion) and clinical (alanine aminotransferase [ALT] increase > 3 × ULN) HBVr in the past HBV selected cohort [14]. Secondary endpoints were characterization of reactivated patients and hematological therapy discontinuation rates.

### 2.3. Data Analysis

The qualitative variables are expressed as counts and percentages, and the discrete variables as median values and interquartile ranges (IQR). Survival was estimated with the Kaplan Meier method and the log rank test was applied to compare the survival distributions of the samples. Statistical analysis was carried out using R v 4.0.0.

## 3. Results

### 3.1. Patients’ Baseline Characteristics

**Figure 1:**
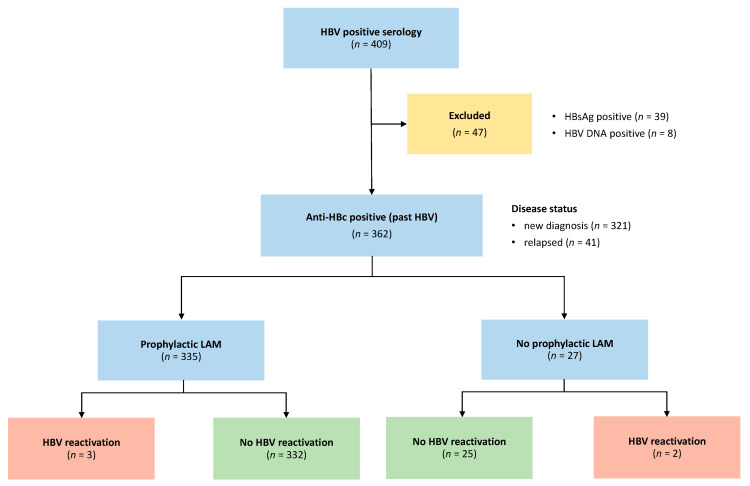
Study flow chart. HBV = Hepatitis B Virus; HBsAg = HBV surface antigen; anti-HBc = antibody to HBV core antigen; LAM = lamivudine.

Before starting chemotherapy, all patients were studied for HBV and HCV serology. Among 409 patients, 47 were excluded due to HBsAg positivity (*n* = 39) or HBV DNA positivity (*n* = 8). These lasts were not included because the study was aimed to potential occult B infection (pOBI—anti-HBc-positive without evidence of detectable viremia) and not to overt occult B infection (OBI) carriers (HBV DNA-positive). Between the 1 January 2007 and the 29 February 2016, 362 patients were enrolled, with last follow-up recorded on the 18 October 2017: a study flow chart is illustrated in Figure 1 and patients baseline characteristics are summarized in Table 1.

Median age at diagnosis was 68 years (range 38-85), with a majority of males (*n* = 198, 55%) and Caucasian ethnicity (*n* = 353, 97.5%). Almost half of the patients were affected by diffuse large B cell lymphoma (DLBCL—*n* = 177, 48.9%), followed by follicular lymphoma (FL—*n* = 78, 21.5%) and mantle cell lymphoma (MCL—*n* = 34, 9.4%). The remaining 73 patients (20.2%) had other types of NHL (mainly lymphoplasmacytic lymphoma [LPL] and marginal zone lymphoma [MZL]). Overall, almost the totality of enrolled patients was treated with a R-containing regimen (*n* = 349, 96.4%), while the remaining received R alone (*n* = 13, 3.6%). The most used immunochemotherapy scheme was an R plus anthracycline regimen (R-CHOP-like—*n* = 212, 58.6%) and nearly 90% of cases (*n* = 321, 88.7%), were receiving their first line of treatment. Thirty-one of 362 (8.6%) patients received an autologous stem cell transplantation (ASCT).

### 3.2. HBV Serology Status and Hepatological Characteristics

Hepatological characteristics of enrolled patients are shown in Table 2. As per study criteria, all were HBsAg-negative and anti-HBc-positive (past HBV). Anti-HBs was negative in 29%, while anti-HBe was positive in 23.5% of cases. Anti-HCV antibodies were detectable in 41 patients (11.4%). Before chemotherapy initiation, ALT levels were >40 U/L in 38 patients (10.6% of available data) and total bilirubin was >1.5 mg/dL in 16 cases (3.4%).

NAT prophylaxis was performed with LAM in 335 of the 362 patients (92.5% of total).

### 3.3. Overall Series Outcome

**Figure 2:**
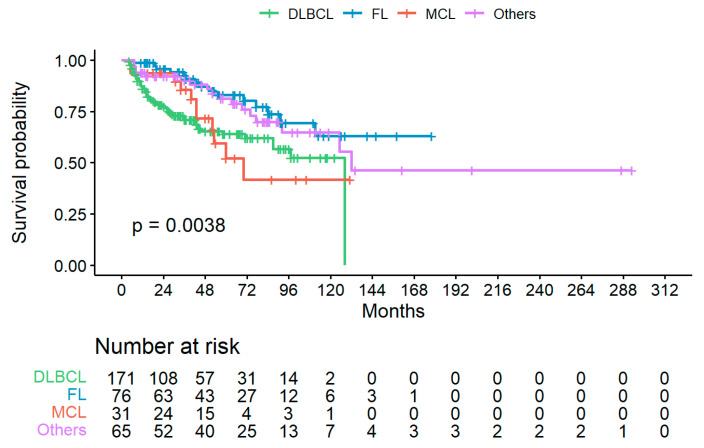
Overall survival (OS) of enrolled patients divided per lymphoma type; landmark analysis is starting from date of diagnosis. Only patients with known status (i.e., alive or death for any cause) at last follow up are reported. DLBCL = diffuse large B cell lymphoma; FL = follicular lymphoma; MCL = mantle cell lymphoma.

Best response after chemotherapy completion for the overall series was complete remission (CR) in 277 (76.5%), partial response (PR) in 55 (15.2%), stable disease (SD) in 10 (2.8%) and progressive disease (PD) in 20 (5.5%) patients, respectively. Median follow-up was 34 months (IQR 16–9) from chemotherapy start, and 23 months (IQR 6–48) from last R dose. Overall survival of enrolled patients is shown in Figure 2.

Median duration of prophylaxis was 18 months (IQR 14–25). Among the 335 treated patients, 160 (47.8%) were still assuming LAM at the last visit and 175 (52.2%) had stopped the antiviral; 167 (95.4%) out of these 175 patients had available data after the discontinuation and were followed-up for a median of 27.7 months (IQR 10.8–54.3). Treatment-related adverse events (AEs) were reported during LAM prophylaxis in three patients (out of 335 treated patients, 0.9%): two cases of skin rash and one of gastrointestinal intolerance (epigastric pain). In all three cases, LAM prophylaxis was interrupted.

### 3.4. HBV Reactivated Patients’ Outcomes

While on LAM prophylaxis, virological and serological but no clinical HBV reactivation was observed in 3 (#1, #3 and #4) out of 335 patients (0.9%). Among those not receiving LAM prophylaxis (*n* = 27), HBVr was noticed in two patients (7.4%): one (patient #2) who developed also a clinical flare, and a further patient (#5) who experienced HBV DNA reactivation without HBsAg seroreversion (this patient was included in the non-prophylaxis group since independently discontinued the drug). Characteristics of reactivated patients are shown in Table 3 and Table 4. Among them, only one patient (patient #3), affected by FL, had already received at least a previous line of treatment for lymphoma.

Among patients not receiving NAT prophylaxis, HBVr occurred 3.5 and 9.9 months (patients #2 and #5, respectively) after the beginning of the R-containing therapy. On the other hand, HBVr was noted 4.5 months after the start of immunochemotherapy in patient #4 (during LAM prophylaxis). The other two subjects (patients #1 and #3) experienced late HBVr, 40.6 months (patient #1) and 34.7 months (patient #3) after the beginning of the R-containing regimen, respectively, and both still receiving LAM prophylaxis. However, while patient #1 concluded the R-containing regimen 35.7 months before HBV reactivation, patient #3 received the last R dose 1.8 months before (R-maintenance in FL).

In the group of LAM prophylaxis (patient #1, #3 and #4) the HBV reactivations were treated with TDF achieving a prompt complete virological response (HBV DNA negative) and normal transaminases. Among the 27 patients of the study not treated with LAM prophylaxis, the virological, serological and clinical reactivation of patient #2 was treated with ETV because not previously exposed to LAM with a complete virological, serological (HBsAg-negative) and clinical response. On the other hand, patient #5, having showed only virological HBVr after LAM withdrawal, simply restarted the drug with benefit. At last follow-up, all hepatitis reactivation events were resolved, and four patients (#2, #3, #4 and #5) were HBsAg-negative. Two patients (#1 and #2) were still receiving antiviral therapy, while the remaining (#3, #4 and #5) discontinued NAT. Of note, all of them maintained the original virological profile of past HBV (HBsAg-negative, anti-HBc-positive, HBV DNA negative) after the antiviral therapy discontinuation. No patient had to interrupt the chemotherapy program due to HBV reactivation, while two patients (#1 and #2) required a delay. Patient #1, affected by MCL, died because of progressive lymphoma disease 2 months after HBV reactivation. In this patient, although HBsAg was still positive, HBV DNA was undetectable at last follow-up.

## 4. Discussion

With 362 enrolled patients, this study represents one of the largest fully annotated cohorts of patients with past HBV treated for lymphoma especially in Western Countries, where incidence of HBV chronic infections and number of past HBV is lower than Asia [1,2].

In this cohort of patients with hematological diseases and a virological profile of past HBV receiving a R-containing regimen, HBVr was a rare event: respectively, 0.8% of cases treated with LAM prophylaxis and 7.4% of those untreated. The overall lower rate of HBVr compared with previous experiences from Asia [8,10] reflects the difference in terms of epidemiology and virological characteristics of the natural history of HBV infection in Europe. However, even if the low rate of HBVr does not allow to define a statistical significance, it confirms an evident higher clinical risk in untreated patients. Moreover, although including both cases at diagnosis and relapse and with no risk stratification, the overall survival of enrolled patients could be reasonably approximated to patients free of past HBV, thus confirming the efficacy of LAM prophylaxis in this setting.

A second important aspect of this study is the very low rate of hepatitis (only one case of clinical flare out of five patients with virological and serological HBVr) and the prompt treatment of all cases. This evidence confirms a high rate of awareness and clinical attention to the risk of HBVr among Italian hematologists, in accordance with a previous report [24]. The attention was probably influenced by the first guidelines endorsed by the Italian Association for the Study of the Liver (AISF) aimed for this setting and published in 2007 [15]. Moreover, this document was preliminary to the authorization of LAM prescription and reimbursement from the Italian Healthcare System for prophylaxis of HBVr in patients with onco-hematological diseases and past HBV infection (HBsAg-negative). The availability of the drug and the simplification of the strategy in patients with past HBV have had a deep impact among hematologists since the most significant rates of HBVr is concentrated in this field and particularly in patients treated with R-containing regimen or undergoing HSCT. Finally, the use of a rational rescue therapy (TDF in LAM-exposed and ETV in naive) confirms the multidisciplinary cooperation between hematologists and hepatologists.

Nevertheless, there is no global consensus on which is the best strategy to avoid HBV reactivation in patients with past HBV. The so called “pre-emptive” strategy, consisting in monthly HBV DNA monitoring, is still proposed by some Authors, especially in Asian Countries [16,25,26]. However, recent data from the phase 3 GOYA and GALLIUM studies reported higher incidence rates of HBV reactivation in patients not receiving prophylactic NAT (10.8% vs 2.1%). In these two trials, almost half of the reactivated patients (12/25) showed a delay in their immunochemotherapy administration; thus, a superiority in the pre-emptive strategy is still not demonstrated [17]. Moreover, the frequent monitoring is complicated and expensive, in terms of both laboratory supplies and staff. Finally, monthly blood collection can be considerably demanding, especially for patients who are out of active treatment for lymphoma or not easily connected to the hospital facilities.

For these reasons, the AISF Italian Guidelines [15,27] and recently an ASCO document [14], confirmed the choice of using HBsAg as a definition of reactivation, and consequently this has become the marker instead of HBV DNA monitoring in case of preemptive strategy. The use of HBsAg is cheaper and is more specific since only 50% of patients with past HBV (anti-HBc-positive) and virological reactivation during the monitoring (HBV DNA-positive) develop the serological reactivation (HBsAg seroreversion), which is the only virological condition constantly associated with the clinical hepatitis [28].

While Western Countries Guidelines converge on the necessity of prophylaxis, there is still debate on which NAT should be used in this subset of patients. The American Gastroenterological Association Institute Guidelines suggest the use of antiviral drugs with a high barrier to resistance over LAM (weak recommendation; moderate-quality evidence); however, they specify that, due to the geographic variability in cost of antiviral therapy, LAM should be preferred in those patients with a negligible risk of resistance development (particularly in those who have an undetectable viral load, a condition constantly associated with past HBV, and/or who are expected to use NAT for ≤6 months) [29].

In addition, the EASL recommends antiviral prophylaxis with NAT in this context (evidence level II-2, grade of recommendation 1) which should continue for at least 18 months after stopping immunosuppression: LAM may be used safely, although few cases of HBV exacerbation due to LAM resistance have been reported and these cases can be effectively treated with a prompt rescue therapy activated before the clinical flare, as reported in our study. Moreover, monitoring for at least 12 months after prophylaxis withdrawal is also recommended, since many studies described a higher risk of HBVr after the discontinuation, rather than during the antiviral therapy [3].

In Italy, the AISF guidelines suggest LAM use in past HBV considered at high risk of HBVr (including HSCT) [11,30], keeping the use of ETV or TDF or TAF in case of positive HBsAg serology (overt HBV infection), serological HBVr (HBsAg-seroreversion in past HBV) or detectable HBV DNA during LAM prophylaxis (virolological breakthrough) [27]. A recent real-life study by the Milan group confirmed the safety profile and cost effectiveness of LAM use in lymphoma patients with past HBV undergoing R-containing immunochemotherapy [19].

To date, no head-to-head randomized controlled studies of LAM vs ETV or TDF are available; only two randomized studies compared ETV or TDF vs no treatment in prophylaxis of HBVr in patients with a virological profile of past HBV. Both studies demonstrated that the risk of HBV reactivation under CT plus Rituximab was of 0% and 2.4% using prophylaxis with TDF or ETV, respectively, significantly lower than without prophylaxis [9,13]. Of interest, in both studies the rate of HBsAg seroreversion (HBsAg-positive) and hepatitis in patients with the virological reactivation (HBV DNA-positive) during the follow-up was only of 50% and 33%, respectively, confirming data previously reported in this discussion.

Our study, with a significantly broad number of patients enrolled from different Italian centers, supports the use of LAM, confirming this universal prophylaxis in patients with past HBV as a safe and effective strategy. Other than the efficacy, demonstrated by the extremely low percentage of HBV reactivation, the low incidence of adverse reactions (0.9% of treated patients) highlighted a very good safety profile of LAM prophylaxis. Moreover, the simplified monitoring of HBsAg (once every 3 months during treatment and over the first year after suspension) together with the low price of LAM, permitted a significant saving of money and human resources to both the local hospital agencies and the National Healthcare Service.

## 5. Conclusions

In conclusion, this study represents one of the largest multicenter cohorts of previously HBV infected patients affected by NHL and treated with R-based chemoimmunotherapy to date. It confirms the safety, efficacy and cost effectiveness of LAM used as prophylaxis during R-containing regimens in these patients if continued until 18 months after the end of immunosuppression, as suggested by national guidelines. HBV reactivation should be monitored with HBsAg conversion every 3 months during therapy and at least one year after the interruption of prophylaxis, together with transaminases and HBV DNA in subjects with evidence of seroreversion (HBsAg-positive) in order to activate a prompt rescue therapy with ETV, TDF or TAF.

## Figures and Tables

**Table 1:** Baseline characteristics of patients with past HBV infection (*n* = 362) *.

**Median Age, Years (Range)**	68 (38–85)
**Gender, *n* (%)**	
Female	164 (45.3)
Male	198 (54.7)
**Ethnicity, *n* (%)**	
Caucasian	353 (97.5)
Asian	4 (1.1)
African	3 (0.8)
South American	2 (0.6)
**Histology, *n* (%)**	
DLBCL	177 (48.9)
FL	78 (21.5)
MCL	34 (9.4)
Others	73 (20.2)
**Treatment regimen, *n* (%)**	
R-CHOP-like	212 (58.6)
R-HDCT-like	40 (11.0)
R-OTHER-like	97 (26.8)
R alone	13 (3.6)
**Disease status**	
Diagnosis	321 (88.7)
Relapse	41 (11.3)
**ASCT, *n* (%)**	31 (8.6)

* DLBCL = diffuse large B cell lymphoma; FL = follicular lymphoma; MCL = mantle cell lymphoma; R = rituximab; CHOP = cyclophosphamide, vincristine, prednisone; HDCT = high dose chemotherapy; ASCT = autologous stem cell transplantation.

**Table 2:** Virological characteristics of patients with past HBV infection (*n* = 362).

	*n* (%)	Missing *
**Anti-HBs**		1.4
Yes	254 (71.1)	
No	103 (28.9)	
**Anti-HBe**		0.3
Yes	85 (23.5)	
No	276 (76.5)	
**Anti-HCV**		0.8
Yes	41 (11.4)	
No	318 (88.6)	
**ALT (U/L)**		0.8
≤40	321 (89.4)	
>40	38 (10.6)	
**Total Bilirubin (mg/dL)**		1.4
≤1.5	345 (96.6)	
>1.5	12 (3.4)	
**Splenomegaly (>13 cm)**		2.5
Yes	78 (22.1)	
No	275 (77.9)	
**Splenectomy**		2.5
Yes	8 (2.3)	
No	345 (97.7)	
**Platelets count (×10^9^/L)**		0.6
<100	34 (9.4)	
≥100	326 (90.6)	

* In the “missing” column percentages (out of 362 total patients) of lacking data for each characteristic are reported (i.e., missing data/overall). HBV = hepatitis B virus; anti-HBs = HBV surface antigen antibody; anti-HBe = hepatitis B e antigen antibody; anti-HCV = hepatitis C virus antibody; ALT = alanine aminotransferase.

**Table 3:** Characteristics of reactivated patients (*n* = 5).

**Patient**	**#1**	**#2**	**#3**	**#4**	**#5**
**Age (years)**	72	55	66	45	59
**Gender**	F	F	F	F	F
**Histology**	MCL	MZL	FL	DLBCL	DLBCL
**Disease Status**	Diagnosis	Diagnosis	Relapse	Diagnosis	Diagnosis
**Treatment regimen**	R-BAC	R-CVP	R-CVP	R-CHOP	R-CHOP
**Disease status at last follow-up**	PD—death	CR	CR	CR	CR
**NAT prophylaxis**	LAM	-	LAM	LAM	-
Timing of reactivation (months) *	40.6	3.5	34.7	4.5	9.9
**HBV reactivation treatment**	TDF	ETV	TDF	TDF	LAM

* Months from beginning of R-chemo. All patients receiving NAT prophylaxis reactivated while were still on treatment with NAT. F = female; MCL = mantle cell lymphoma; MZL = marginal zone lymphoma; FL = follicular lymphoma; DLBCL = diffuse large B cell lymphoma; R-BAC = rituximab, bendamustine, cytarabine; R-CVP = rituximab, cyclophosphamide, vincristine, prednisone; R-CHOP = rituximab, cyclophosphamide, vincristine, prednisone; PD = progressive disease; CR = complete remission; LAM = lamivudine; ETV = entecavir; TDF = tenofovir disoproxil fumarate.

**Table 4:** Hepatological characteristics of reactivated patients (*n* = 5).

	Basal			Reactivation			Last Follow-Up		
Patient *	Anti-HBs	Anti-Hbe	ALT	HBsAg	HBV DNA	ALT	HBsAg	HBV DNA	ALT
**#1**	Positive	Negative	15	Positive	136108	8	Positive	Negative	21
**#2**	Negative	Negative	NA	Positive	NA	492	Negative	Negative	13
**#3**	Positive	Negative	11	Positive	570	11	Negative	Negative	18
**#4**	Negative	Positive	12	Positive	<12	18	Negative	Negative	NA
**#5**	Positive	Negative	35	Negative	463	27	Negative	Negative	35

* Patients #2 and #5 were not receiving LAM prophylaxis. HBV = hepatitis B virus; anti-HBs = anti-HBV surface antigen antibody; anti-HBe = hepatitis B e antigen antibody; ALT = alanine aminotransferase; HBsAg = HBV surface antigen; NA = not available.

## Data Availability

The data presented in this study are available on request from the corresponding author.
